# Bacterial symbiosis in arthropods and the control of disease transmission.

**DOI:** 10.3201/eid0404.980408

**Published:** 1998

**Authors:** C B Beard, R V Durvasula, F F Richards

**Affiliations:** Centers for Disease Control and Prevention, Atlanta, Georgia 30333, USA. cbb0@cdc.gov

## Abstract

Bacterial symbionts may be used as vehicles for expressing foreign genes in arthropods. Expression of selected genes can render an arthropod incapable of transmitting a second microorganism that is pathogenic for humans and is an alternative approach to the control of arthropod-borne diseases. We discuss the rationale for this alternative approach, its potential applications and limitations, and the regulatory concerns that may arise from its use in interrupting disease transmission in humans and animals.


### Bacterial Symbiosis in Arthropods and the Control of Disease Transmission

**Charles B. Beard,* Ravi V. Durvasula,† and Frank F. Richards†**
*Centers for Disease Control and Prevention, Atlanta, Georgia, USA; and
†Yale University School of Medicine, New Haven, Connecticut, USA

Bacterial symbionts may be used as vehicles for expressing foreign genes in arthropods. Expression of selected genes can render an arthropod incapable of transmitting a second microorganism that is pathogenic for humans and is an alternative approach to the control of arthropod-borne diseases. We discuss the rationale for this alternative approach, its potential applications and limitations, and the regulatory concerns that may arise from its use in interrupting disease transmission in humans and animals.

For more than 80 years, insecticides have been one of the primary means of controlling insect-borne diseases. Recently, however, the control of insect pests and vectors of disease has become increasingly difficult for various reasons, including the emergence of insecticide resistance, changes in the environment, and reduction in public health interventions due to social and economic problems in countries where insect-borne diseases are endemic. According to the World Health Organization (WHO), approximately 125 arthropod species are resistant to at least one, and often two or more, insecticides (1). In many parts of the world where insect-borne diseases cause illness and death, insecticides are available; however, sustaining insecticide vector control long term can be extremely costly and may be unachievable.

### Alternative Approaches to Controlling Disease Transmission by Arthropods

Concerns related to the use of insecticides for vector control have led to alternative approaches for reducing disease transmission by arthropods. One approach focuses on the use of transgenic methods, i.e., the insertion and expression of a gene derived from one organism in a second, heterologous organism. Major components of the transformation system include the identification of 1) potential DNA vectors (transposable elements or viral-transducing agents) for genome integration (2-8); 2) selectable phenotypic marker genes, such as eye color mutants or various enzymes (e.g., ß-galactosidase, neomycin phosophotransferase, or organophosphate-degrading enzyme), as indicators of stable germ line transformation (9-11); and 3) specific refractory genes that express the desired phenotype (i.e., a factor that would inhibit transmission of the pathogen) (12-14). Additional studies have focused on the identification of regulatory sequences (e.g., stage-specific, tissue-specific, and constitutive promoters [15-20]), vector population genetics (21-23), and the development of mathematical models that can be used for predicting the behavior of genes once they are introduced into wild populations (24,25). The accomplishments and future prospects of efforts to produce transgenic arthropods have been discussed extensively (26-31).

Another approach for reducing disease transmission by arthropods is to genetically modify symbiotic bacteria of arthropod vectors to prevent the arthropods from transmitting human pathogens. With this approach, the arthropod is not transformed, but the symbiotic bacteria that it harbors are (32). Such arthropods are called paratransgenic. This approach is guided by the following basic concepts: 1) many arthropods (especially those that throughout their entire developmental cycle feed on restricted food sources such as blood, cellulose, phloem, stored grains) harbor bacterial symbionts; 2) in some cases, these symbionts can be

Address for correspondence: Charles B. Beard, Centers for Disease Control and Prevention, 1600 Clifton Road, Mail Stop F12, Atlanta, GA 30333, USA; fax: 770-488-7469; e-mail: cbb0@cdc.gov.

cultured and genetically transformed to express a gene whose product kills a pathogen that the arthropod transmits; 3) normal arthropod symbionts can be replaced with genetically modified symbionts, resulting in a population of arthropod vectors that can no longer transmit disease. While not applicable to all groups of arthropods, this approach has worked in three species of Chagas disease vectors and holds promise in a number of other arthropods.

### Bacterial Symbionts and Control of American Trypanosomiasis

American trypanosomiasis (Chagas disease) is a parasitic illness that affects 16 to 18 million people in regions of South and Central America, according to current WHO estimates. Neither a cure for chronic Chagas disease nor a vaccine for preventing infection is available. The etiologic agent, the flagellate protozoon *Trypanosoma cruzi*, is transmitted by blood-sucking triatomine bugs, which become infected while feeding on an infected host. Transmission to humans occurs as the insect feeds when the engorged triatomine bug, while feeding, deposits on the skin a fecal droplet that contains infective trypanosomes, which then get rubbed either into the bite lesion or a mucous membrane (Figure 1).

*Rhodnius prolixus* is the chief vector of Chagas disease in certain regions of Central America and northern South America. *Rhodococcus rhodnii*, a soil-associated nocardiform actinomycete, residing extracellularly in the gut lumen of *R. prolixus* in close proximity to *T. cruzi* (33), is transmitted effectively from adult triatomid bugs to their progeny through coprophagy (ingestion of fecal material from other bugs). The vital role of *R. rhodnii* in the growth and development of *R. prolixus* has been demonstrated repeatedly under laboratory conditions (34-36). Aposymbiotic nymphs of *R. prolixus* (insects that have been cured of symbionts) do not reach the sexually mature adult stage; most deaths occur after the second developmental molt. Introduction of the bacteria to first or second instar nymphs permits normal growth and maturation.

Scientists have exploited this symbiotic association to introduce and express a series of foreign gene constructs in *R. prolixus* (37,38). DNA shuttle plasmids capable of replication in both *Escherichia coli* and in *R. rhodnii* have been constructed and used to genetically modify *R. rhodnii* (Figure 2). The genetically altered symbionts can then be introduced into aposymbiotic first instar nymphs of *R. prolixus,* where, like unmodified microorganisms, they allow normal growth and reproduction of the insect while expressing specific gene products of interest.

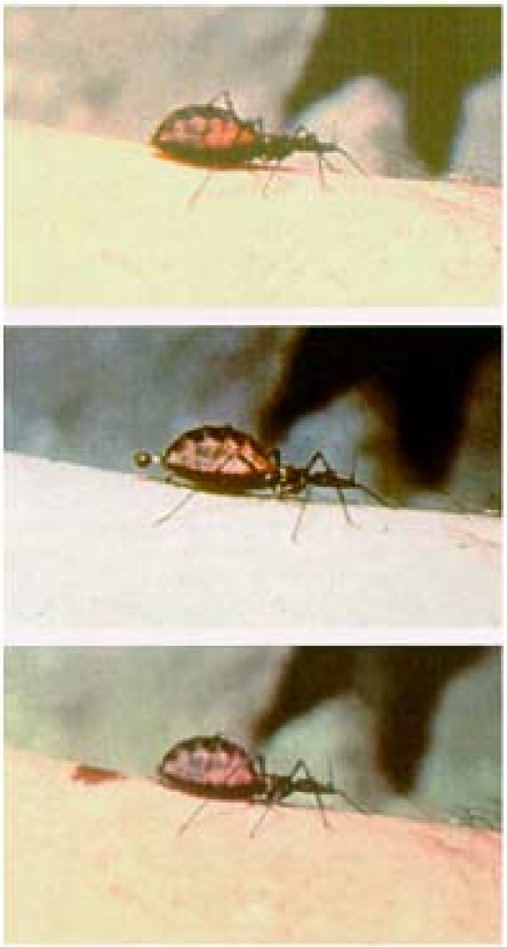

Figure 1. A triatomine bug vector of Chagas disease in the process of feeding. The fecal droplet contains infective trypanosomes and bacterial symbionts. (Photographs courtesy of Robert B. Tesh).

In our laboratory, a selectable genetic marker coding for resistance to the antibiotic thiostrepton was expressed by transgenic symbionts within the gut of insects colonized experimentally (37). The insects could be fed on blood that contained thiostrepton, and the bacteria survived and persisted throughout the insect's development to adulthood. Furthermore, we showed that when thiostrepton was omitted from the blood, the symbionts maintained

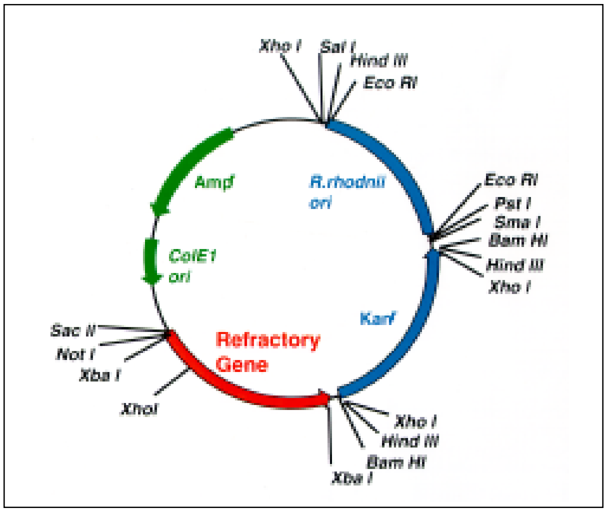

Figure 2. Shuttle plasmid for genetic transformation of *Rhodococcus rhodnii*.

resistance to the antibiotic, which indicates that the plasmid was stable in its bacterial host. By sterilizing the surface of the eggs with a topical iodine solution and colonizing the resulting aposymbiotic (sterile) insects with genetically modified symbionts (delivered to the insects orally), we developed a line of paratransgenic arthropods in which individual insects were refractory to infection with *T. cruzi* (38). Refractoriness was conferred after the antimicrobial peptide L-Cecropin A was expressed and secreted. The gene for this peptide was contained in a shuttle plasmid expression vector used to transform *R. rhodnii,* which subsequently expressed and secreted the gene product within the gut of experimentally colonized insects (38). Cecropin A, a 38–amino acid peptide, belongs to a family of small channel-forming peptides with potent antimicrobial activity; these peptides insert into biologic membranes, forming channels that ultimately lead to cell lysis and death (39-41). This family of peptides has been isolated from several insect and vertebrate tissues, which they defend against bacterial pathogens (41). Cecropin A has strong lytic activity against *T. cruzi* but negligible deleterious effects on *R. rhodnii* or gut tissues of *R. prolixus*. Laboratory colonies of *R. prolixus* that carried genetically altered *R. rhodnii* transformed to express mature Cecropin A were completely refractory to infection with *T. cruzi* strain DM28 in approximately 70% of the insects. In the remaining insects, numbers of *T. cruzi* were reduced to less than 1% of the numbers seen in control *R. prolixus*, which carried untransformed *R. rhodnii*.

These studies demonstrate that symbiotic bacteria of disease-transmitting triatomine bugs can be genetically modified to express biologically active molecules and then can be reintroduced into the host insect, expressing the desired phenotype (Figure 3). The genetically altered *R. rhodnii* allowed normal development and sexual maturation of the host insect.

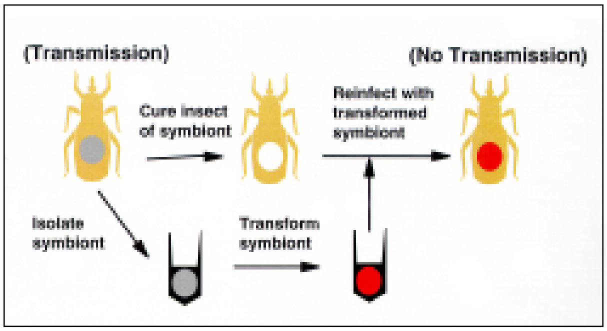

Figure 3. Symbionts can be genetically altered and used to replace native symbionts, resulting in insects that can no longer transmit disease. (Illustration courtesy of Mark Q. Benedict).

### Delivery Mechanisms/Field Applications

For the bacterial symbiont approach to be used in a field intervention program, a mechanism must be developed for spreading the genetically altered bacteria through an insect population. The delivery system must allow dispersal of recombinant genetic material without adverse environmental effects. Using bacteria that have specialized symbiotic associations with specific insect hosts to spread transgenes greatly reduces the chance of unwanted gene spread; the natural insect-symbiont association can be used for this purpose. In *R. prolixus,* early instar nymphs acquire the symbiont *R. rhodnii* by coprophagy. When they first emerge, instar nymphs are transiently aposymbiotic; they pick up the required bacteria by probing the eggshell or fecal droplets of other bugs (Figure 1). Scientists have observed that triatomine bugs actively probe small dots of black ink on paper, apparently because they resemble the black fecal droplets shed by triatomine bugs after digestion of the bloodmeal. When live bacterial cultures and a small amount of India ink are added to an inert carrier, a formulation of the genetically modified bacteria is produced that resembles bug feces and can be ingested by immature insects; in this way, modified bacteria can be established uniformly and effectively in laboratory colonies of *R. prolixus* (37,38). Studies to test this approach in the laboratory use a design that simulates a field application. Wooden frames composed of housing materials common in the rural tropics (primarily mud and thatch) are treated with the bacterial formulation. Field-collected *R. prolixus* are added to these frames, which are then enclosed in large plexiglass containers. The progeny of the field-collected insects are examined for colonization with the modified symbiont, ability of the symbiont to compete with native symbionts established in the field-collected specimens, and expression of the desired transmission phenotype.

A possible strategy for using vector-symbiont intervention for the control of Chagas disease transmission would require that in disease-endemic areas, individual houses likely to become infested with triatomines be treated with the bacterial formulation, either when they are new and uncolonized or after insecticide treatment that kills any resident insects, especially in corners and cracks where they are most likely to hide. In homes treated with the bacterial formulation, which likely contain wild-type symbionts, the genetically modified symbionts could be given a competitive advantage of being applied in greater concentrations than the native symbionts. As triatomine bugs from adjacent untreated areas reinfest the house, they would lay their eggs, which would hatch within days. The digestive tract of the new immature bugs would be colonized through coprophagy with genetically altered symbionts, which would keep them from subsequently becoming infected with the Chagas disease trypanosome. These progeny would then amplify and disperse the altered symbionts through natural coprophagic routes. Since triatomine bugs involved in domiciliary transmission must invade and become established in the homes, peridomestic or sylvatic habitats would not need to be treated to affect domestic transmission. Because of the labor and expense involved in repeated insecticide treatment, reinfestation is a potential major obstacle in Chagas disease control. Vector-symbiont intervention could play an important role in an integrated control approach that used a combination of insecticidal and molecular genetic interventions.

### Bacterial Symbionts in Tsetse

Bacterial symbionts have also been evaluated in other insect disease vectors. The tsetse vectors of African sleeping sickness (trypanosomiasis) harbor as many as three distinct populations of bacterial flora (33,42-45). Like triatomine bugs, tsetse are obligate bloodfeeders, and at least one population of bacteria (found in uterine secretory cells) is presumed to be nutritional mutualist symbionts. The primary, or P-symbionts, are highly specialized intracellular bacteria apparently essential for the flies' survival (33,45). These bacteria have not been cultured or transformed. The secondary, or S-symbionts, comprise another population of gram-negative bacteria found in various tissues of tsetse, including the salivary glands, where they reside in large numbers, especially in older flies (S. Aksoy, pers. comm.). S-symbionts can be isolated from hemolymph and cultivated in vitro, where they have been shown to grow both intra- and extracellularly (Figure 4) (46-48). A potential transformation system has been developed for these bacteria with the recombinant plasmid pSUP204 (47). This DNA vector contains the broad host range replicon *oriV,* derived from a *Pseudomonas aeruginosa* plasmid, RSF1010, and ligated into the *E. coli* cloning vector pBR325 (49,50). Recent studies indicate that genetically transformed tsetse S-symbionts can be microinjected into recipient flies and express a reporter

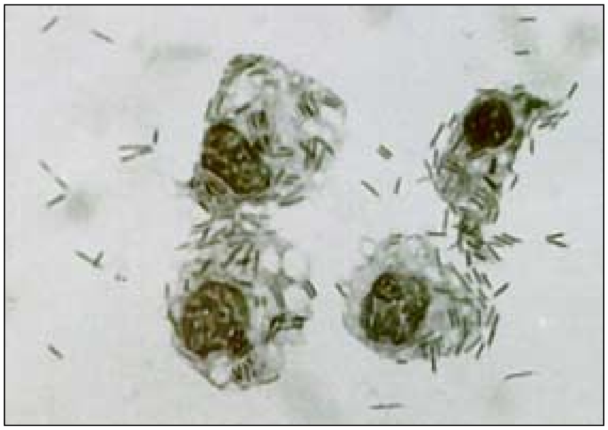

Figure 4. The tsetse secondary symbiont GP01 growing intracellularly and extracellularly in culture.

gene (S. Aksoy, pers. comm.). This area of research will likely yield new approaches for controlling tsetse transmission of trypanosomes.

### Intracellular Insect Reproductive System Agents—*Wolbachia*

Obligate intracellular bacteria found in many species of arthropods (51,52), the *Wolbachia* are maternally transmitted from parent to offspring and are often involved in a variety of reproductive anomalies, such as cytoplasmic incompatibility (reproductive incompatibility due to maternal, nongenetic factors), sex ratio determination and distortions, and parthenogenesis (53-56). Although extremely fastidious, these microbes could be used in the transgenic alteration of arthropod vectors in two ways (32): 1) direct transformation and subsequent expression of a gene product by the *Wolbachia* in the arthropod, and 2) use of *Wolbachia*-induced cytoplasmic incompatibility to drive a second, maternally inherited factor into a population of vectors. The first approach entails the use of either episomal plasmids or DNA integration vectors for transformation of the *Wolbachia*. Although theoretically possible, such a transformation system (i.e., a suitable DNA vector and methods for introducing the DNA and selecting for transformants) does not now exist. Recent successful genetic transformation of rickettsial agents, however, suggests that this limitation may soon be overcome (57).

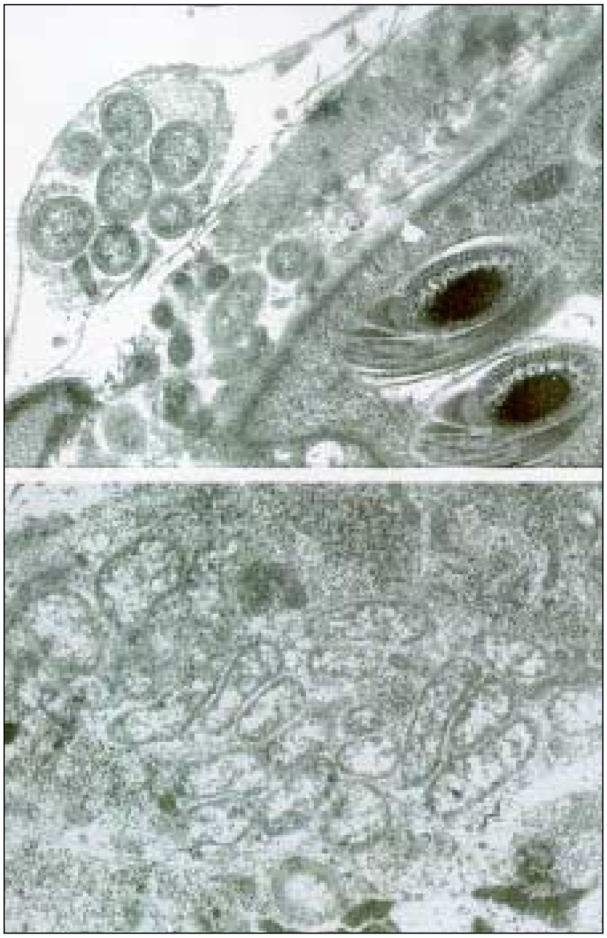

Figure 5. *Wolbachia*-like organisms in insect reproductive tissues.

Since *Wolbachia* are frequently observed in reproductive tissues of arthropods (Figure 5), one potential approach that they might be used to disrupt transmission of a pathogen is suggested by studies performed using a viral transduction system in the mosquito *Aedes aegypti* (14). In these experiments, antisense DNA sequences corresponding to a membrane protein of the dengue type 2 virus were expressed somatically by using a recombinant Sindbis virus vector. Mosquitoes that were coinfected with the two viruses were shown not to be able to transmit dengue virus. Similar work has been done with *Aedes triseriatus* mosquitoes, which are potential vectors of the LaCrosse virus (58). These types of experiments could be done by using genetically modified *Wolbachia*, with the goal of blocking transovarial transmission of an arboviral agent, such as La Crosse encephalitis virus or Rift Valley Fever virus, which is dependent on vertical transmission from the adult mosquito to its progeny for maintenance of the virus in nature. Genetically transformed *Wolbachia* could be utilized in a manner similar to that of viral transducing agents to express antisense DNA sequences that interfere with replication of transovarially transmitted virus pathogen.

The disruption of transovarial transmission is an application that could potentially be adapted for use in a number of species of mosquitoes, ticks, and mites in which transovarial transmission of pathogens is important. Very recent studies report the distribution of *Wolbachia* throughout somatic and germ-line tissues in various insects. These observations suggest that potential transmission-blocking applications using genetically modified *Wolbachia* would not be limited simply to transovarially transmitted agents but potentially applicable to microbial pathogens that reside and/or replicate in other insect tissues as well, such as the hemolymph or salivary glands (59).

Alternatively, *Wolbachia* could be used to render a population of arthropod disease vectors incapable of transmitting a disease agent: *Wolbachia*-mediated cytoplasmic incompatibility could spread a second maternally inherited gene into the vector population. In its simplest form (Figure 6), cytoplasmic incompatibility results when a *Wolbachia*-negative female mates with a *Wolbachia*-positive male; no offspring are produced from such an incompatible cross. Since the reciprocal cross between an infected female and an uninfected male results in *Wolbachia*-infected progeny, the net effect in matings between the two strains, other things being equal, is that *Wolbachia*-infected progeny will be more prevalent than *Wolbachia*-free progeny. This phenomenon has been observed in natural populations of *Wolbachia*-infected and -uninfected *Drosophila simulans* in California, demonstrating the rapid spread of *Wolbachia* and the corresponding cytoplasmic incompatibility phenotype across broad geographic regions (61). How this phenomenon might be utilized has been discussed in great detail (32,60).

As *Wolbachia* spread through the population by cytoplasmic incompatibility, other maternally inherited organisms (or organelles) in the *Wolbachia*-infected strains are transmitted. "Cage studies," using mosquitoes with different mitochondrial haplotypes, have found that haplotype frequencies changed so quickly that within two generations, one haplotype was completely replaced by the other as a result of *Wolbachia*-driven cytoplasmic incompatibility (62).

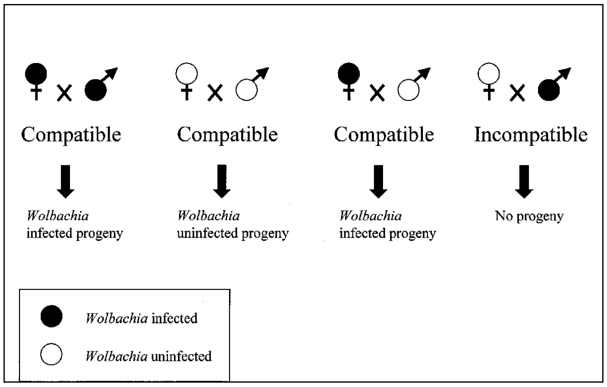

Figure 6. *Wolbachia*-mediated cytoplasmic incompatibility.

In an actual scheme for genetic modification of a vector population, the gene blocking pathogen transmission could be carried by a second, nonpathogenic species of bacteria, such as the tsetse S-symbiont (32,63) or a rickettsialike commensal that is maternally inherited (64), or even possibly an organelle such the mitochondrion. The transformed agent could be introduced into a *Wolbachia*-infected laboratory colony, and the females could be released. Over time, because of the driving mechanism of cytoplasmic incompatibility, a gene product critical for transmission of an arthropod-borne disease agent could be blocked by a second product expressed and secreted by the genetically transformed, maternally inherited microorganism, resulting in the modification of the vector population's capacity to transmit the disease agent.

### Limitations

The application of these methods is limited primarily by the occurrence of suitable microorganisms in specific arthropod vectors. In general, many arthropods (e.g., ticks, mites, triatomine bugs, bed bugs, lice, some species of fleas, and tsetse) that feed on single food sources throughout their entire developmental cycle contain microbial symbionts that produce nutritional supplements lacking in the restricted diet (so called nutritional mutualists) (33,65,66). Mosquitoes and many other dipterans, however, do not fall into this category because they do not feed on blood strictly as a source of nutrition for basal metabolism; rather, the females alone feed on blood, which is used directly to produce progeny. Although other symbionts may be present in mosquitoes, nutritional mutualists are altogether lacking.

Reported from numerous and diverse species of arthropods (51), *Wolbachia* may occur naturally in more than 15% of all insect species (67). *Wolbachia* have been transferred in the laboratory from one arthropod species to another, successfully conferring the cytoplasmic incompatibility phenotype to the recipient species (68,69). Other "passenger" microorganisms, such as tsetse S-symbionts (33,42) and *Rickettsia*-like organisms (70,71), are common in arthropods, and although the nature of their symbiotic relationship with the arthropod host is often unclear, their consistent association suggests their potential as vehicles for expressing a transgene in the arthropod.

Other limitations to the use of arthropod bacteria include the development of appropriate transformation methods and reagents. Because many nonpathogenic microorganisms in arthropods have been poorly studied and characterized, accurate identification of the agent is a prerequisite to genetic transformation studies. These microorganisms can be characterized either through traditional bacteriologic characterization or by a polymerase chain reaction–based approach that uses common eubacterial primers to amplify the 16S rRNA locus that can be sequenced and used for identifying the microbe (72,73). DNA vectors and transformation methods have been developed for diverse groups of both gram-positive and -negative microorganisms; these tools and approaches can often be adapted for use with a bacterial symbiont or passenger. We have efficiently transformed several species of coryneform bacteria, common agents of various triatomine bugs that transmit Chagas disease, by using transformation vectors and regulatory sequences developed for use in *Mycobacterium* spp.

### Release of Paratransgenic Insects: Regulations and Safety

In addition to questions regarding science and efficacy, numerous safety and regulatory concerns pertaining to laboratory and field research with genetically modified arthropod vectors of human disease must be addressed. These concerns include potential environmental and ecologic hazards associated with release; potential public health risks; and overall public perception, which can determine the future of entire programs (74,75).

In the United States, federal guidelines and regulations address a broad range of issues pertaining to research, transport, and release of microorganisms, plants, and insects. The U.S. Department of Agriculture Animal and Plant Inspection Service (APHIS) regulates the release of transgenic arthropods of agricultural importance. None of these guidelines or regulations, however, directly address genetically modified arthropod vectors of human disease agents. Nevertheless, the work that has been done and the policy that is in place set a precedent.

The basic principles of containment regarding research with transgenic and paratransgenic insect disease vectors are similar to those regarding human pathogens and biologic insecticide agents (insects and microorganisms). Guidelines that discuss established standards for work with human disease agents, typically classified as biosafety level (BSL)-2, BSL-3, or BSL-4, on the basis of the assessment of virulence and transmissibility have been published (76), and a permitting process oversees the import and transport of these agents into and throughout the United States. The Environmental Protection Agency (EPA) and USDA-APHIS share oversight with regard to the development and use of biologic insecticides (77). USDA-APHIS also regulates the use of insects and arthropods that are predators of insect pests. The containment procedures and facilities associated with the use of these organisms in new geographic regions in the United States might be adapted as the minimum standards for work with transgenic arthropods (75).

With respect to regulation of the release of transgenic arthropods of agricultural importance and risk assessment, USDA-APHIS has formed an interagency "virtual" committee charged with the responsibility of evaluating proposals to release transgenic arthropods that could affect plants and animals (excluding humans); efforts are under way to form an interagency committee with the authority to regulate the release of arthropods that could affect humans. Information on the program that APHIS has assembled, including details regarding permits and current applications that have been submitted under several plant pest statutes, can be accessed at http://www.aphis.usda.gov/biotech/arthropod/; risk assessment information is also available at this site.

Assessment of the environmental risks of releasing transgenic and paratransgenic arthropods shares principles with environmental impact analysis of biologic insecticides (78). The same arguments used to promote the potential benefits of recombinant organisms are equally effectively used to argue the potential hazards (79). To assess risk, Hoy (74) addresses four basic concerns: 1) the attributes of the unmodified organism; 2) attributes of the genetic alteration, i.e., only of the gene transferred; 3) phenotype of the modified organism compared with the unmodified organism; and 4) attributes of the accessible environment. These concerns can guide formulation of research plans to assess environmental risks associated with the release of transgenic arthropods.

Transgenic and paratransgenic research with arthropods raises several public health concerns. Because the arthropods may transmit human disease agents, ethical issues must be considered. For example, consider that a transgenic or paratransgenic insect is released under the assumption that its ability to transmit disease has been demonstrated to be significantly decreased or eliminated, as determined by laboratory studies in carefully defined conditions. Then suppose that when pilot field studies are conducted, for some reason, it is shown that under true field conditions the ability to transmit disease is not reduced as much as had been anticipated on the basis of the controlled laboratory studies. The result is that the human population present where the release takes place could actually be at increased risk for disease due to the release of large numbers of competent or partially competent vectors. The assessment of this type of risk is extremely important, as is the related human subjects issue of informed consent and how it would be obtained in a study where the population that is potentially at risk cannot be accurately determined.

Public perception, frequently not considered until a potential product is ready for field testing, is extremely important (e.g., public response to genetic control studies in India in the 1970s, including the release of sterile mosquitoes [80] and to the ongoing controversy surrounding the labeling of foods that are, or contain, the products of genetic engineering in agriculture [81-83]). The most effective biotechnology programs are those in which the public is informed and brought into discussions at the earliest possible time (75,84).

The efficacy of a biologic agent to prevent or reduce the impact of a disease is of paramount importance, yet most of this information can be obtained definitively only after the method has been tested on a large scale and under different environmental conditions. On the other hand, information on safety, as well as on the behavior of the agent in the field, is required before we know how well it works and what other effects it has on the environment. Thus, the scientist developing a new agent must make an honest, imaginative leap into the future and try to predict any possible dangerous consequences—the responsibility for risk assessment must be shouldered by the scientist, together with the appropriate regulatory agencies. Because the safety questions are numerous, those of the greatest potential significance, both in the short term and in the long term, should be examined first. The risks associated with the intervention must be assessed against the risk for disease, the risk to health and the environment from pesticides, and the risk of doing nothing.

### Conclusions

Decreasing effectiveness of controlling diseases transmitted by arthropods has led to evaluation of new control strategies, including the use of transgenic methods. A strategy that relies on producing paratransgenic arthropods has been applied with initial success to the triatomine bugs that transmit Chagas disease, resulting in vector strains incapable of being infected with the pathogenic agent *T. cruzi*. This approach may also prove feasible for a number of other vector-borne diseases, using either nutritional symbionts of arthropods that feed exclusively on blood, *Wolbachia* that have been successfully transferred between species in the laboratory, or other microbial passengers consistently associated with their arthropod host. As the tools and methods that allow broader applications of this approach are developed, and actual products arrive at the point of field testing, permitting and regulation will be required.

The regulatory process must begin with the individual investigators, who must thoroughly evaluate the product being developed and work with regulatory agencies and others to ensure that if and when genetically modified organisms are used for the control of insect borne diseases, they will be both safe and efficacious.

### Acknowledgments

The authors thank Drs. Orrey P. Young, Suzanne Binder, and Mark Benedict, for their critical review of this manuscript.

Dr. Beard is research entomologist and chief, Vector Biology Activity, Division of Parasitic Diseases, CDC. Research in his laboratory focuses on the molecular biology of insect disease vectors and the molecular epidemiology of opportunistic infections in persons with AIDS.

### References

1. World Health Organization. Vector resistance to pesticides. 15th report of the WHO Expert Committee on Vector Biology and Control. World Health Organ Tech Rep Ser 1992;818:62.
2. Spradling AC, Rubin GM. Transposition of cloned *P* elements into *Drosophila* germ line chromosomes. Science 1982;218:341-47.
3. Presnail JK, Hoy MA. Stable genetic transformation of a beneficial arthropod, *Metaseiulus occidentalis* (Acari: Phytoseiidae), by a microinjection technique. Proc Natl Acad Sci U S A 1992;89:7732-6.
4. O'Brochta DA, Warren WD, Saville KJ, Atkinson PW. Interplasmid transposition of *Drosophila hobo* elements in non-drosophilid insects. Mol Gen Genet 1994;244:9-14.
5. O'Brochta DA, Warren WD, Saville KJ, Atkinson PW. *Hermes*, a functional non-drosophilid insect gene vector from *Musca domestica*. Genetics 1996;142:907-14.
6. Loukeris TG, Livadaras I, Arca B, Zabalou S, Savakis C. Gene transfer into the Medfly, *Ceratitis capitata*, using a *Drosophila hydei* transposable element. Science 1995;270:2002-5.
7. Coates CJ, Jasinskiene N, Miyashiro L, James AA. *Mariner* transposition and transformation of the yellow fever mosquito, *Aedes aegypti*. Proc Natl Acad Sci U S A 1998;95:3748-51.
8. Jasinskiene N, Coates CJ, Benedict MQ, Cornel AJ, Rafferty CS, James AA, et al. Stable transformation of the yellow fever mosquito, *Aedes aegypti*, with the *Hermes* element from the housefly. Proc Natl Acad Sci U S A 1998;95:3743-7.
9. Klemenz R, Wever U, Gehring WJ. The white gene as a marker in a new P-element vector for gene transfer in *Drosophila*. Nucleic Acids Res 1987;15:3947-59.
10. Beard CB, Benedict MQ, Primus JP, Finnerty V, Collins FH. Eye pigments in wild-type and eye-color mutant strains of the African malaria vector *Anopheles gambiae*. J Hered 1995;86:375-80.
11. Benedict MQ, Salazar CE, Collins FH. A new dominant selectable marker for genetic transformation; *Hsp 70-opd*. Insect Biochem Mol Biol 1995;25:1061-5.
12. Collins FH, Sakai RK, Vernick KD, Paskewitz SM, Seeley DC, Miller LH, et al. Genetic selection of a *Plasmodium*-refractory strain of the malaria vector *Anopheles gambiae*. Science 1986;234:607-10.
13. Vernick KD, Collins FH, Gwadz RW. A general system of resistance to *Plasmodium* infection in the vector *Anopheles gambiae* is controlled by two main genetic loci. Am J Trop Med Hyg 1989;40:585-92.
14. Olson KE, Higgs S, Gaines PJ, Powers AM, Davis BS, Kamrud IK, et al. Genetically engineered resistance to dengue-2 virus transmission in mosquitoes. Science 1996;272:884-6.
15. James AA, Blackmer K, Marinotti O, Ghosn CR, Racioppi JV. Isolation and characterization of the gene expressing the major salivary gland protein of the female mosquito *Aedes aegypti*. Mol Biochem Parasitol 1991;44:245-54.
16. Müller HM, Crampton JM, della Torre A, Sinden R, Crisanti A. Members of a trypsin gene family in *Anopheles gambiae* are induced in the gut by blood meal. EMBO J 1993;12:2891-900.
17. Salazar CE, Hamm DH, Wesson DM, Beard CB, Kumar V, Collins FH. A cytoskeletal actin gene in the mosquito *Anopheles gambiae*. Insect Mol Biol 1994;3:1-13.
18. Benedict MQ, Scott JA, Cockburn AF. High-level expression of the bacterial opd gene in *Drosophila melanogaster*: improved inducible insecticide resistance. Insect Mol Biol 1994;3:247-52.
19. Benedict MQ, Levine BJ, Ke ZX, Cockburn, AF, Seawright JA. Precise limitation of concerted evolution to ORFs in mosquito *Hsp82* genes. Insect Mol Biol 1996;5:73-9.
20. Beard CB, Cornel AJ, Collins FH. The polyubiquitin gene of the mosquito *Anopheles gambiae*: structure and expression. Insect Mol Biol 1996;5:109-17.
21. Guhl F, Schofield CJ. Population genetics and control of triatominae. Parasitology Today 1996;12:169-70.
22. Besansky NJ, Lehmann T, Fahey GT, Fontenille D, Braack LE, Hawley WA, et al. Patterns of mitochondrial variation within and between African malaria vectors, *Anopheles gambiae* and *An. arabiensis*, suggest extensive gene flow. Genetics 1997;147:1817-28.
23. Lehmann T, Besansky NJ, Hawley WA, Fahey TG, Kamau L, Collins FH. Microgeographic structure of *Anopheles gambiae* in western Kenya based on mtDNA and microsatellite loci. Mol Ecol 1997;6:243-53.
24. Kidwell MG, Ribeiro JMC. Can transposable elements be used to drive disease refractoriness genes into vector populations? Parasitology Today 1992;8:325-9.
25. Ribeiro JMC, Kidwell MG. Transposable elements as population drive mechanisms: specification of critical parameter values. J Med Entomol 1994;33:10-6.
26. Eggleston P. The control of insect-borne disease through recombinant DNA technology. Heredity 1991;66:161-72.
27. Warren AM, Crampton, JM. *Mariner*: its prospects as a DNA vector for the genetic manipulation of medically important insects. Parasitology Today 1994;10:58-63.
28. Carlson JO, Olson KE, Higgs S, Beaty BJ. Molecular genetic manipulation of mosquito vectors. Annu Rev Entomol 1995;40:359-88.
29. O'Brochta DA, Atkinson PW. Transposable elements and gene transformation in non-drosophilid insects. Insect Biochem Mol Biol 1996;26:739-53.
30. O'Brochta DA, Atkinson PW. Recent developments in transgenic insect technology. Parasitology Today 1997;13:99-104.
31. Collins FH, Paskewitz SM. Malaria: current and future prospects for control. Annu Rev Entomol 1995;40:195-219.
32. Beard CB, O'Neill SL, Tesh RB, Richards FF, Aksoy S. Modification of arthropod vector competence via symbiotic bacteria. Parastiology Today 1993;9:179-83.
33. Dasch GA, Weiss E, Chang K. Endosymbionts of insects. In: Krieg NR, editor. Bergey's manual of systematic bacteriology. Vol. 1. Baltimore: Williams & Wilkins; 1984. p. 811-33.
34. Baines S. The role of the symbiotic bacteria in the nutrition of *Rhodnius prolixus* (Hemiptera). J Exp Biol 1956;33:533-41.
35. Harrington JS. Studies on *Rhodnius prolixus*: growth and development of normal and sterile bugs, and the symbiotic relationship. Parasitology 1960;50:279-86.

36. Hill P, Campbell JA, Petrie IA. *Rhodnius prolixus* and its symbiotic actinomycete: a microbiological, physiological and behavioural study. Proc R Soc Lond B Biol Sci 1976;194:501-25.
37. Beard CB, Mason PW, Aksoy S, Tesh RB, Richards FF. Transformation of an insect symbiont and expression of a foreign gene in the Chagas' disease vector *Rhodnius prolixus*. Am J Trop Med Hyg 1992;46:195-200.
38. Durvasula RV, Gumbs A, Panackal A, Kruglov O, Aksoy S, Merrifield RB, et al. Prevention of insect-borne disease: an approach using transgenic symbiotic bacteria. Proc Natl Acad Sci U S A 1997;94:3274-8.
39. Christensen B, Fink J, Merrifield RB, Mauzerall D. Channel-forming properties of cecropins and related model compounds incorporated into planar lipid membranes. Proc Natl Acad Sci U S A 1988;85:5072-6.
40. Wade D, Boman A, Wahlin B, Drain CM, Andreu A, Boman H, et al. All-D amino acid-containing channel-forming antibiotic peptides. Proc Natl Acad Sci U S A 1990;87:4761-5.
41. Boman HG. Antibacterial peptides: key components needed in immunity. Cell 1991;65:205-7.
42. O'Neill SL, Gooding RH, Aksoy S. Phylogenetically distant symbiotic microorganisms reside in *Glossina* midgut and ovary tissues. Med Vet Entomol 1993;7:377-83.
43. Aksoy S. *Wigglesworthia* gen. nov. and *Wigglesworthia glossinidia* sp. nov., taxa consisting of the mycetocyte-associated, primary endosymbionts of tsetse flies. Int J Sys Bacteriol 1995;45:848-51.
44. Aksoy S, Pourhosseini AA, Chow A. Mycetome endosymbionts of tsetse flies constitute a distinct lineage related to Enterobacteriaceae. Insect Mol Biol 1995;4:15-22.
45. Southwood TRE, Khalaf S, Sinden RE. The micro-organisms of tsetse flies. Acta Trop 1975;32:259-66.
46. Welburn SC, Maudlin I, Ellis DS. *In vitro* cultivation of rickettsia-like-organisms from *Glossina* spp. Ann Trop Med Parasitol 1987;81:331-5.
47. Beard CB, O'Neill SL, Mason P, Mandelco L, Woese CR, Tesh RB, et al. Genetic transformation and phylogeny of bacterial symbionts from tsetse. Insect Mol Biol 1993;1:123-31.
48. Welburn SC, Dale C. Isolation and culture of tsetse secondary endosymbionts. In: Crampton JM, Beard CB, Louis C, editors. Molecular biology of insect disease vectors: a methods manual. London: Chapman & Hall; 1997. p. 547-54.
49. Priefer UB, Simon R, Puhler A. Extension of the host range of *Escherichia coli* vectors by incorporation of RSF1010 replication and mobilization function. J Bacteriol 1985;163:324-30.
50. Reshcke DK, Frazier ME, Mallavia LP. Transformation of *Rochalimaea quintana*, a member of the family Rickettsiaceae. J Bacteriol 1990;172:5130-4.
51. O'Neill SL, Giordano R, Colbert AME, Karr TL, Robertson HM. 16S rRNA phylogenetic analysis of the bacterial endosymbionts associated with cytoplasmic incompatibility in insects. Proc Natl Acad Sci U S A 1992;89:2699-702.
52. O'Neill SL. *Wolbachia pipientis*: symbiont or parasite. Parasitology Today 1995;11:168-9.
53. Hoffmann AA, Turelli M. Cytoplasmic incompatibility in insects. In: O'Neill SL, Hoffmann A, Werren J, editors. Influential passengers. Oxford: Oxford University Press; 1997. p. 42-80.
54. Rigaud T. Inherited microorganisms and sex determination of arthropod hosts. In: O'Neill SL, Hoffmann A, Werren J, editors. Influential passengers. Oxford: Oxford University Press; 1997. p. 81-101.
55. Stouthamer R. *Wolbachia*-induced parthenogenesis. In: O'Neill SL, Hoffmann A, Werren J, editors. Influential passengers. Oxford: Oxford University Press; 1997. p. 102-22.
56. Hurst GDD, Hurst LD, Majerus EN. Cytoplasmic sex-ratio distorters. In: O'Neill SL, Hoffmann A, Werren J, editors. Influential passengers. Oxford: Oxford University Press; 1997. p. 125-54.
57. Rachek LI, Tucker AM, Winkler HH, Wood DO. Transformation of *Rickettsia prowazekii* to rifampin resistance. J Bacteriol 1998;180:2118-24.
58. Powers AM, Kamrud KI, Olson KE, Higgs S, Carlson JO, Beaty BJ. Molecularly engineered resistance to California serogroup virus replication in mosquito cells and mosquitoes. Proc Natl Acad Sci U S A 1996;93:4187-91.
59. Dobson SL, Bourtzis K, Braig HR, Jones BF, Weigou Z, O'Neill SL. *Wolbachia* infections are distributed throughout insect somatic and germ line tissues. Insect Biochem Mol Biol. In press 1998.
60. Sinkins SP, Curtis CF, O'Neill SL. The potential application of inherited symbiont systems to pest control. In: O'Neill SL, Hoffmann A, Werren J, editors. Influential passengers. Oxford: Oxford University Press; 1997. p. 155-75.
61. Turelli M, Hoffmann AA. Rapid spread of an inherited incompatibility factor in California *Drosophila*. Nature 1991;353:440-2.
62. Kambhampati S, Rai KS, Verleye DM. Frequencies of mitochondrial DNA haplotypes in laboratory cage populations of the mosquito, *Aedes albopictus*. Genetics 1992;132:205-9.
63. Beard CB, Aksoy S. Genetic manipulation of insect symbionts. In: Crampton JM, Beard CB, Louis C, editors. Molecular biology of insect disease vectors: a methods manual. London: Chapman & Hall; 1997. p. 555-60.
64. Carl M. Genetic characterization and transovarial transmission of a typhus-like rickettsia found in cat fleas. Proc Natl Acad Sci U S A 1992;89:43-6.
65. Buchner P. Endosymbiotis of animals with plant microorganisms. New York: Interscience; 1965.
66. Douglas AE, Beard CB. Midgut symbionts. In: Lehane MJ, Billingsley PF, editors. Biology of the insect midgut, London: Chapman & Hall; 1996. p. 419-31.
67. Werren JH, Windsor D, Guo LR. Distribution of *Wolbachia* among neotropical arthropods. Proc Royal Soc Lond B Biol Sci 1995;262:197-204.
68. Boyle L, O'Neill SL, Robertson HM, Karr TL. Interspecific and intraspecific horizontal transfer of *Wolbachia* in *Drosophila*. Science 1993;260:1796-9.
69. Braig HR, Guzman H, Tesh RB, O'Neill SL. Replacement of the natural *Wolbachia* symbiont of *Drosophila simulans* with a mosquito counterpart. Nature 1994;367:453-5.

70. Werren JH, Hurst GD, Zhang W, Breeuwer JAJ, Stouthamer R, Majerus MEN. Rickettsial relative associated with male killing in the ladybird beetle (*Adalia bipunctata*). J Bacteriol 1994;176:388-94.
71. Azad AF, Beard CB. Rickettsial pathogens and their arthropod vectors. Emerg Infect Dis 1998;4:179-86.
72. Higgins JA, Azad AF. Use of the polymerase chain reaction to detect bacteria in arthropods: a review. J Med Entomol 1995;32:213-22.
73. O'Neill SL. PCR-based detection and identification of insect symbionts. In: Crampton JM, Beard CB, Louis C, editors. Molecular biology of insect disease vectors: a method manual. London: Chapman & Hall; 1997. p. 561-6.
74. Hoy MA. Impact of risk analyses on pest-management programs employing transgenic arthropods. Parasitology Today 1995;11:229-32.
75. Hoy MA. Laboratory containment of transgenic arthropods. Amer Entomol 1997;43:206-56.
76. Biosafety in microbiology and biomedical laboratories. 3rd ed. Atlanta (GA): Centers for Disease Control and Prevention; 1993. Report No.: 93-8395.
77. Shantharam S, Foudlin AS. Federal regulation of biotechnology: jurisdiction of the U.S. Department of Agriculture. In: Maramorosch K, editor. Biotechnology for biological control of pests and vectors. Boca Raton (FL): CRC Press; 1991. p. 239-50.
78. Hollander AK. Environmental impacts of genetically engineered microbial and viral biocontrol agents. In: Maramorosch K, editor. Biotechnology for biological control of pests and vectors. Boca Raton (FL): CRC Press; 1991. p. 251-66.
79. Simonsen L, Levin BR. Evaluating the risk of releasing genetically engineered organisms. Tree 1988;3:s27.
80. Curtis CF, von Borstel RC. Allegations against Indian research unit refuted. Nature 1978;273:96.
81. UK inquiry into genetic engineering of crops. Nature 1998;392:534.
82. Consumers unhappy with food regulations. Nature 1997;389:657.
83. Wadman M. EPA to be sued over gene-modified crops. Nature 1997;389:317.
84. Asner M. Public relations: the scientist and the public, the government, and the media. In: Purchase HG, MacKenzie, editors. Agricultural biotechnology. Introduction to field testing. Washington: Office of Agricultural Biotechnology, USDA; 1990. p. 35.
